# Comparison of a point-of-care analyser for the determination of HbA1c with HPLC method

**DOI:** 10.1016/j.plabm.2017.04.001

**Published:** 2017-04-04

**Authors:** D.A. Grant, G.J. Dunseath, R. Churm, S.D. Luzio

**Affiliations:** Diabetes Research Unit Cymru, Grove Building, Swansea University, Swansea SA2 8PP, UK

**Keywords:** POCT, Point Of Care Testing, HPLC, High Performance Liquid Chromatography, HbA1c, Glycated haemoglobin, Point of care testing, HbA1c measurement

## Abstract

**Aims:**

As the use of Point of Care Testing (POCT) devices for measurement of glycated haemoglobin (HbA1c) increases, it is imperative to determine how their performance compares to laboratory methods. This study compared the performance of the automated Quo-Test POCT device (EKF Diagnostics), which uses boronate fluorescence quenching technology, with a laboratory based High Performance Liquid Chromatography (HPLC) method (Biorad D10) for measurement of HbA1c.

**Methods:**

Whole blood EDTA samples from subjects (n=100) with and without diabetes were assayed using a BioRad D10 and a Quo-Test analyser. Intra-assay variation was determined by measuring six HbA1c samples in triplicate and inter-assay variation was determined by assaying four samples on 4 days. Stability was determined by assaying three samples stored at −20 °C for 14 and 28 days post collection.

**Results:**

Median (IQR) HbA1c was 60 (44.0–71.2) mmol/mol (7.6 (6.17–8.66) %) and 62 (45.0–69.0) mmol/mol (7.8 (6.27–8.46) %) for D10 and Quo-Test, respectively, with very good agreement (R^2^=0.969, P<0.0001). Mean (range) intra- and inter-assay variation was 1.2% (0.0–2.7%) and 1.6% (0.0–2.7%) for the D10 and 3.5% (0.0–6.7%) and 2.7% (0.7–5.1%) for the Quo-Test. Mean change in HbA1c after 28 days storage at −20 °C was −0.7% and +0.3% for D10 and Quo-Test respectively. Compared to the D10, Quo-Test showed 98% agreement for diagnosis of glucose intolerance (IGT and T2DM) and 100% for diagnosis of T2DM.

**Conclusion:**

Good agreement between the D10 and Quo-Test was seen across a wide HbA1c range. The Quo-Test POCT device provided similar performance to a laboratory based HPLC method.

## 1 Introduction

In the past, the recommended method for the diagnosis of diabetes was through the repeated measurement of fasting plasma glucose or an oral glucose tolerance test (OGTT) [1]. Although widely used, these methods can be time-consuming for both the patient and the clinical team. More recently, the measurement of the glycated fraction (A1c) of haemoglobin (HbA1c) has been recommended to diagnose diabetes [2], in addition to monitoring glycaemic control. HbA1c reflects long-term glycaemic control over the preceding 2–3 months as opposed to glycaemic control at a single point in time e.g. fasting plasma glucose [3].

HbA1c is a fundamental measure for the assessment of diabetes control, with elevated levels of ≥48 mmol/mol (≥6.5%) being the recommended criterion for diabetes diagnosis, 42–47 mmol/mol (6.0–6.4%) for impaired glucose tolerance (IGT) and <42 mmol/mol (<6.0%) for normal glucose tolerance (NGT) [4], [5]. Point of Care Testing (POCT) enables measurement of HbA1c in a clinical setting without the waiting time associated with laboratory testing or the high level of expertise required; however, for POCT methods to be used effectively they must be comparable to those used in a laboratory.

The Quo-Test is a POCT analyser using boronate fluorescence quenching technology for the measurement of HbA1c traceable to the IFCC reference method. The analyser runs a single sample, requiring no user intervention following sample loading.

The aim of this study was to compare the performance of the Quo-Test POCT analyser with a HPLC laboratory method.

## 2 Materials and methods

HbA1c was determined using the BioRad D10 HPLC analyser (Biorad, Hemel Hempstead, UK) as the laboratory reference method and the Quo-Test POCT analyser (EKF, Penarth, UK). The BioRad D10 required 10 μL and the Quo-Test 4 μL of whole blood. Quality control was carried out every day of testing following the manufacturer's instructions using Biorad Lyphocheck Haemoglobin A1c Controls for the D10 and EKF Diagnostics Quo-Test A1c Controls for the Quo-Test.

### 2.1 Method precision

Precision was determined by calculating the mean coefficient of variation (%CV) from six HbA1c samples assayed in triplicate for intra-assay variation and from repeat assaying of four samples on four days for inter-assay variation (range 34–72 mmol/mol for BioRad D10 and 33–104 mmol/mol for Quo-Test).

### 2.2 Method comparison

Whole blood EDTA samples (n=100) from subjects with and without diabetes were collected and assayed between June and August 2015. Ethical approval was obtained from the South East Wales Research Ethics Committee.

### 2.3 Stability

To test the stability of whole blood samples, aliquots from three samples were frozen immediately post measurement on day zero and stored at −20 °C. Thawed aliquots were measured on days 14 and 28 using both methods. The mean change in HbA1c over 28 days and the %CV (0, 14 and 28 day values) were calculated.

## 3 Results

### 3.1 Method precision

Mean (range) intra- and inter-assay CV for the D10 was 1.2% (0.0–2.7%) and 1.6% (0.0–2.7%), respectively and for the Quo-Test was 3.5% (0.0–6.7%) and 2.7% (0.7–5.1%), respectively.

### 3.2 Method comparison

**Fig. 1:**
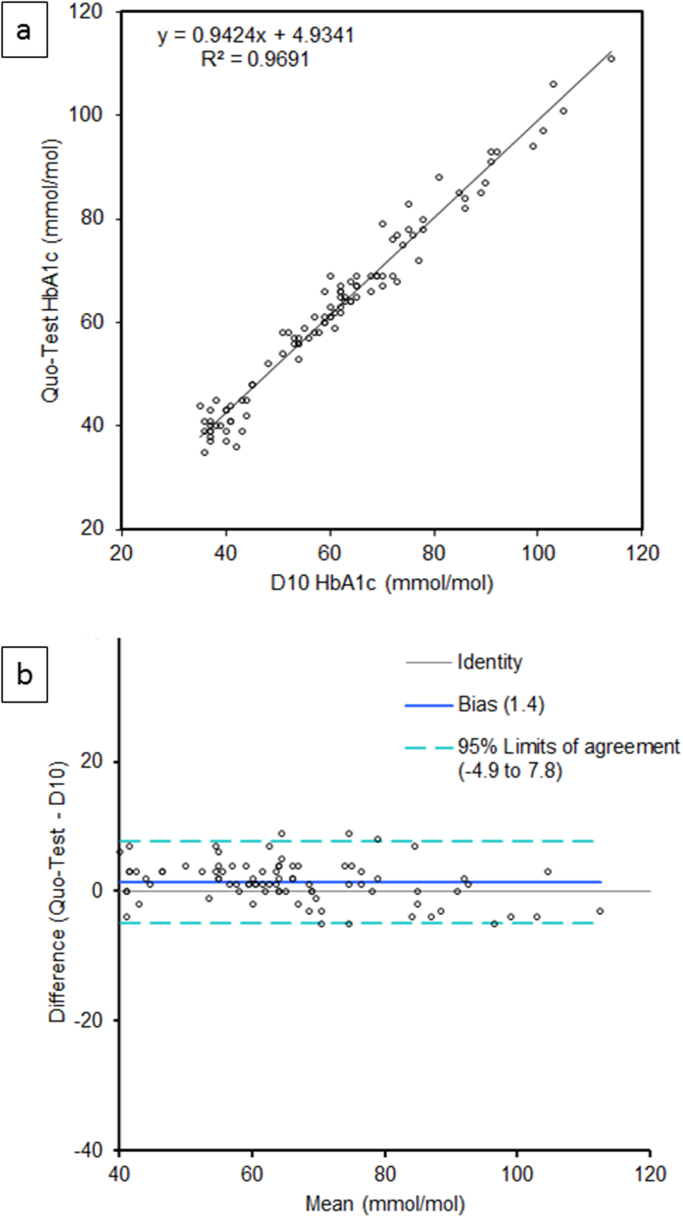
a) Method comparison and b) difference plot for D10 vs. Quo-Test.

The comparison between D10 and Quo-Test is shown in Fig. 1. The median (IQR) HbA1c concentration was 60 (44.0–71.2) mmol/mol (7.6 (6.17–8.66) %) and 62 (45.0–69.0) mmol/mol (7.8 (6.27–8.46) %) for D10 and Quo-Test, respectively with a mean difference (2 SD) of 1.4 (6.4) mmol/mol. Good overall agreement was observed (R^2^=0.9691; p<0.0001).

### 3.3 Stability

**Fig. 2:**
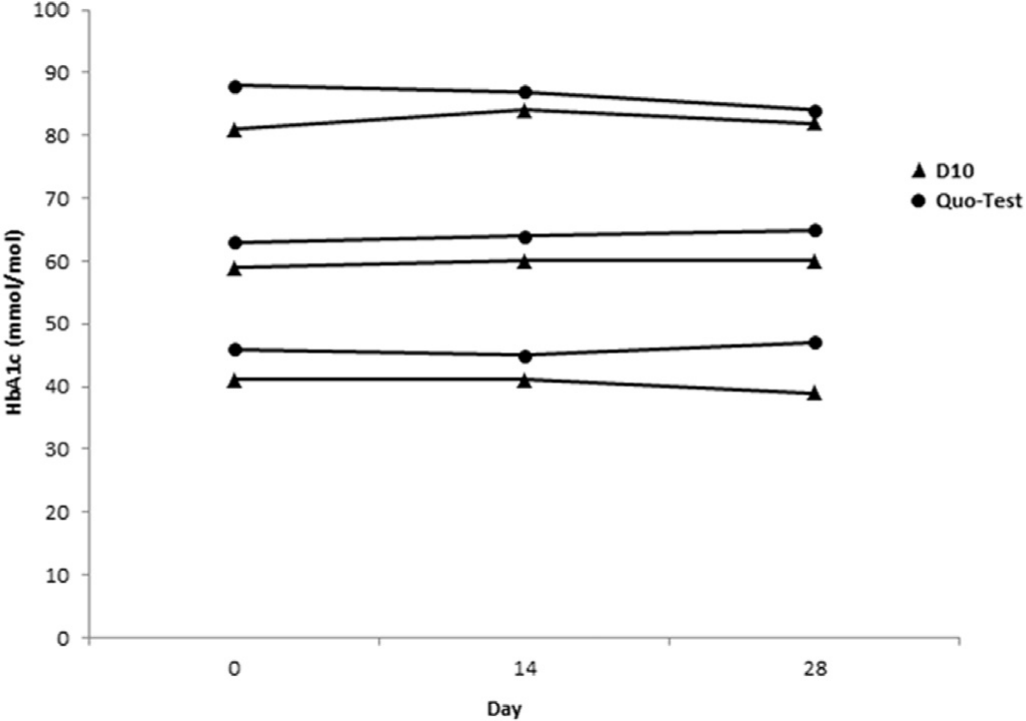
Sample stability (n=3) after short term (28 days) storage at −20 °C.

Thawed aliquots that were measured on day 14 and 28 using both methods showed good stability. Over the period of 28 days the mean change in concentration from baseline for 3 samples was −0.7% (−4.9%, +1.7% and +1.2%) for the D10, and +0.3% (+2.2%, +3.2% and −4.5%) for the Quo-Test (Fig. 2). Mean %CV for the 0, 14 and 28 day samples were 1.9% and 2.0% for the D10 and Quo-Test, respectively, therefore the %CV for the Quo-Test was within the observed inter-assay variation.

### 3.4 Diagnostic comparison

According to WHO diagnostic ranges for HbA1c [4], samples were categorised as being glucose intolerant (≥42 mmol/mol; i.e. both impaired glucose tolerant and T2DM) or T2DM (≥48 mmol/mol). Use of the Quo-test as a diagnostic tool, showed 97% and 100% agreement respectively for the samples classified by the D10 as glucose intolerant (n=79) or T2DM (n=72). Only 2 of the 79 subjects with glucose intolerance and none of the 72 subjects with diabetes would have been under-classified by the Quo-Test when using the D10 result as the laboratory analyser confirmation.

## 4 Discussion

The purpose of this study was to compare the Quo-Test POCT analyser with an established laboratory method.

Overall, there was very good agreement between the D10 reference analyser and the Quo-Test. The Quo-Test also gave results comparable to those given by the D10 after short term storage of whole blood samples at −20 °C. Previous studies have shown sample stability after short and long term storage at −70 °C for the measurement of HbA1c [6] but this study has shown that storage for up to 28 days at −20 °C is acceptable for measurement on the Quo-Test and D10 alike.

The Quo-Test, as a POCT analyser, has limitations compared to an HPLC method such as the inability to measure HbA1c outside the working range of 20–140 mmol/mol 4–15%). Also, as with any other HbA1c analyser, the result obtained using the Quo-Test is dependent on the patient's clinical history and may be affected in patients who have an abnormal typical lifespan of their blood cells, for example haemolytic anaemia [7] which results in a lower HbA1c reading, independent of glycaemic control. Because of the boronate fluorescence quenching technology used the Quo-Test does not detect abnormal haemoglobins and may not give an accurate result relative to glucose, however the Quo-Test may be less affected than HPLC by haemoglobin variants which do not shorten erythrocyte lifespan. The Quo-Test allows the reliable measurement of HbA1c within minutes in a clinical setting, allowing for a real-time therapeutic decision for management of diabetes whilst the patient is present without the delay associated with sending samples off to a laboratory for testing. This is ideal for settings that do not have access to a laboratory and does not incur the large cost and required training associated with laboratory analysers.

Despite the improvements in performance of POCT compared to laboratory analysers, the use of POCT HbA1c measurement in diagnosis of diabetes is not currently supported in clinical guidelines [6]. For POCT to be considered for diagnostic purposes in clinical guidelines, POCT analysers would need to be operated by trained laboratory personnel, be part of a recognised external quality assurance scheme and be able to demonstrate comparable analytical quality to that of laboratory analysers. Any diagnosis of diabetes would subsequently need to be confirmed by a laboratory analyser [8].

## 5 Conclusions

The performance of the Quo-Test POCT analyser is similar to that of a laboratory HPLC analyser. The Quo-Test POCT analyser should be considered for diagnostic purposes by the various professional organisation that issue clinical guidelines.
